# New insights of potential biomarkers in diabetic retinopathy: integrated multi-omic analyses

**DOI:** 10.3389/fendo.2025.1595207

**Published:** 2025-07-01

**Authors:** Xurui Wang, Weina Liu, Xiujin Zheng, Ming Ming Yang

**Affiliations:** ^1^ Department of Ophthalmology, Shenzhen People’s Hospital (The First Affiliated Hospital, Southern University of Science and Technology, The Second Clinical Medical College, Jinan University), Shenzhen, China; ^2^ Department of Ophthalmology, South China Hospital, Shenzhen University, Shenzhen, China

**Keywords:** diabetic retinopathy, omics, genomics, transcriptomics, proteomics, metabolomics, biomarkers

## Abstract

Diabetes mellitus prevalence is rising worldwide, with a predicted 20% increase between 2021 and 2030, bringing an increased burden of complications such as diabetic retinopathy (DR). DR is a common and serious ocular complication of diabetes, and one of the most common irreversible blinding ophthalmic diseases. Its pathogenesis is intricate and complex, involving hypoxia, oxidative stress, inflammation, abnormalities in the polyol metabolic pathway, and others. Clinical detection of DR is impeded by atypical early symptoms, imperfect imaging screening tools, ocular comorbidities (e.g., cataract), and shortages of human resources. Therefore, more in-depth studies are needed to improve DR diagnosis and identify higher-risk patients. “Omics” encompasses genomics, transcriptomics, proteomics, and metabolomics. Omics technologies are increasingly used in research seeking to identify biomarkers or early preclinical signs of disease, or to better understand complex pathological processes determining disease prognosis. And DR is no exception, as an area in need of improved understanding and prognosis. To date, research has yielded significant results advancing DR diagnosis and treatment, informing prevention strategies and reducing global disease impact. This article reviewed recent findings of omics in DR diagnosis and treatment, improving our understanding of DR pathology and enabling personalized treatments.

## 1 Introduction

Diabetes mellitus is a group of metabolic diseases characterized by hyperglycemia, and its prevalence has been increasing worldwide, resulting in a serious social burden (1, 2). Diabetes mellitus can cause a variety of macrovascular and microvascular complications (3), including ocular complications such as diabetic retinopathy (DR), metabolic cataract, arterial nerve injury, ocular inflammation, refractive error, etc. Among them, DR is considered the most common and damaging ocular complication, affecting a wide range of people with poor prognosis, currently the focus of research and a hot topic in ophthalmology. It affects a wide range of people and has a poor prognosis, currently the focus of research and a hot topic in ophthalmology. The pathogenesis of DR is intricate and complex, currently believed to be initiated by prolonged exposure to hyperglycemia and other causative risk factors (e.g., hypertension) that induce a series of physiological and biochemical changes within the eye, including oxidative stress, protein kinase C (PKC) activation, inflammation, and abnormalities in polyol metabolism pathways, ultimately leading to microvascular injury and retinal dysfunction (4, 5). Current treatments cannot prevent DR onset and can only delay it to a small extent, emphasizing the need for better treatments, ideally started early, to prevent onset or delay deterioration in high-risk individuals. Frequent retinal screening of all diabetic patients is an effective way to prevent DR complications; however, many patients have ocular comorbidities such as cataracts that impede clinical diagnosis (6). This distinction relies on specialized ophthalmologists; however, due to the shortage of eye care human resources, a major challenge in the coming years will be better predicting patients at risk of developing vision-threatening DR stages (7).

Routine screening for diabetic retinopathy (DR) effectively prevents DR and enables early treatment to minimize vision loss. However, there are no specific biomarkers for diagnosing the early onset and progression of DR. Multi-omics offers an exceptional opportunity to advance molecular understanding of eye disease, including how environmental, social, economic, and cultural exposures affect molecular eye health. Multi-omics is crucial for overcoming eye health inequalities. This article reviews recent advances in multi-omics research for DR, employing approaches that comprehensively understand pathophysiologic mechanisms and biomarkers, as well as deepen understanding of associated biochemical pathways, which may provide future therapeutic targets.

## 2 Application of histologic analysis techniques in DR

### 2.1 Multi-omics based approach for analyzing biological samples

In biological mechanisms, the basic flow of information in biological systems is from DNA (genome) to RNA (transcriptome) to proteins (proteome) to metabolites (metabolome). “omics” strategies are designed to investigate millions of markers that simultaneously represent similar biochemical identities by providing high-throughput interfaces. These techniques can be used to discover the underlying molecular properties present behind complex phenotypes (8). The types of data available for omics technologies include genomics, transcriptomics, proteomics, and metabolomics, among others. While somatic mutations certainly occur (and are critical in cancer), from the perspective of ocular disease, DNA sequences can be viewed as largely static and consistent throughout the body, in contrast to the proteome and metabolome, which are highly dynamic. Indeed, the proteome and metabolome provide biological information at a specific time and place, which is closer to the disease phenotype.

Large-scale genomics studies (including GWAS, WES, etc.) can help us discover hundreds of DR-related gene loci; however, these gene loci, which are located upstream of biological mechanisms, are still a long way from becoming useful therapeutic targets. Methodology, engineering, and equipment design have advanced dramatically over the past few decades, and the number of biological samples that can be analyzed using high-throughput genomics methods has increased significantly. Significant advances in mass spectrometry (MS) technology have enabled more in-depth analyses and identification of a greater number of proteins at a faster rate (9, 10), and the application of these studies has been extended to ophthalmic research. Therefore, there is a need to integrate the results of multi-omics studies or conduct systematic studies to gain a more comprehensive understanding of the pathogenesis and therapeutic targets of DR.

Systems medicine is an approach to studying complex metabolic pathways that involves analyzing the entire “ome” of organisms, including the genome, transcriptome, proteome, and metabolome (11, 12). This differs from the traditional approach of focusing on individual biomarkers because it evaluates the entire array of intermediary metabolisms and assesses the quantitative relationships between these metabolites. While this doesn’t necessarily produce biologically interpretable single numbers, it does enable the analysis of three-dimensional maps or patterns of metabolites.

Omics technology has different levels of integrated studies for a particular disease, and human diseases are composed of complex biological processes and diverse metabolic pathways that have a molecular basis for interaction and are influenced by environmental factors (13). However, these studies are relatively simple to analyze, and multi-omics technology can help achieve a holistic view by simultaneously studying different omics information in a multidimensional network (14). This can aid in developing knowledge of the molecular basis of potential interactions to more accurately determine disease pathophysiology and its longitudinal effects, potentially providing new avenues for prevention, early diagnosis, and treatment.

### 2.2 Genomics

Genomics, a significant branch of biology, focuses on the study of genome structure, function, evolution, and interactions of organisms, and serves as the foundation of multi-omics disciplines. Numerous genetic studies have demonstrated a clear genetic predisposition to diabetic retinopathy. Family members with diabetic retinopathy (DR) are 2–3 times more likely to develop the disease compared to family members without DR (15). The genetic contribution to DR and proliferative DR (PDR) has been estimated to be as high as 27% and 52%, respectively (16).

#### 2.2.1 Genetic association analysis and candidate gene studies

Early genetic studies of DR primarily utilized Genetic Linkage Analysis and Candidate gene association studies, but these approaches yielded few strong genetic signals (17, 18). Association analysis is effective for single-gene disorders but less so for multifactorial ones like DR, as it provides limited and inconsistent information on potential genetic loci. This approach has largely been supplanted by other genetic analysis methods (19). Candidate gene studies have identified several genes associated with DR, most notably vascular endothelial growth factor (VEGF), which has been closely tied to neovascularization and DR pathogenesis. Other genes of interest include aldose reductase ALR (AKR1B1) (19), erythropoietin (EPO) (20), transcription factor 7-like 2 (TCF7L2) (21), RAGE (receptor of AGEs), and renin-angiotensin system genes (22, 23). However, candidate gene studies have proven unsuccessful due to their insufficient sample size, incomplete variant coverage, and inaccurate assumptions. Such studies are now waning.

#### 2.2.2 Genome-wide association study

Sequencing technologies for genomics mainly include genome-wide association study (GWAS) and whole exon sequencing (WES) technologies. GWAS evaluates genetic variants, generally SNPs, across the genome that may be associated with the disease of interest, and is an unbiased approach covering the entire genome compared to candidate gene analysis. Genome-wide analysis, particularly GWAS, has had considerable success in other complex diseases such as age-related macular degeneration (AMD), type 2 diabetes (T2D) and diabetic nephropathy (24–30). This review summarizes recent genome-wide association analysis studies of diabetic retinopathy (DR) (**Table 1**). An early version of a genome-wide genotyping platform was used in a study of Mexican-American patients with type 2 diabetes, analyzing 103 patients with moderate to severe non-proliferative diabetic retinopathy (NPDR) and proliferative diabetic retinopathy (PDR) versus 183 controls without DR or early NPDR. No detected SNPs reached genome-wide significance levels (31). The second study, of Caucasian type 1 diabetes (T1D) patients, included 973 cases with severe retinopathy defined by laser treatment history, and 1,856 controls untreated for DR. No loci reached genome-wide significance, but several candidate loci were proposed for independent validation (32, 33). Several studies in subsequent years have identified variants that are significantly associated with the entire genome, but further validation is still needed (34–36).

**Table 1:** Summary of genome-wide association studies for diabetic retinopathy.

Study; Platform	Determination of DR	DM	Case; Population	Control; Population	Significant findings (Gene; P-value)
Fu et al. *J Ophthal* 2010 (31); Affymetrix 100K	Bilateral seven standard field photography	2	Moderate to severe NPDR and PDR; Mexican-Americans: 103	No DR to early NPDR; Mexican-Americans: 183	*TINAG*; P=1.8 x 10^−5^ *C6orf170*; P = 2.8 × 10^−5^
Grassi et al. *Hum Mol Genet* 2011 (33); Affymetrix 5.0 and Illumina HapMap550	Questionnaire about history of laser treatment for DR	1	PDR and DME; Caucasian; 973	NPDR and no DR; Caucasian; 1856	*AKT3/ZNF238*; P = 1.2 × 10^−7^ *A2BP1*; P = 6.4 × 10^−7^
Huang et al. *Ophthalmology* 2011 (34); Illumina HumanMap550	Fundus examination and photography	2	NPDR and PDR; Chinese(Taiwanese): 174	No DR and nondiabetic patients; Chinese(Taiwanese): 575	*C5orf36*; P = 3.0 x 10^-15^ *HS6ST3*; P = 4.7 x 10^−11^
Sheu et al. *Hum Mol Genet* 2013 (35); Illumina OmniExpress	Fundus examination	2	PDR ; Chinese: 437	No DR with diabetes for at least 8 years ; Chinese: 570	*TBC1D4-COMMD6*; P = 1.3 × 10 −^7^ *LRP2-BBS5*; P = 2.0 × 10^−6^
Awata T et al. *PLoSOne* 2014 (36); Affymetrix GeneChip 6.0	Fundus exam and international clinical diabetic retinopathy or macular edema disease severity scale	2	DR; Japanese: 837	DM2 w/o DR; Japanese: 1,149	*RP1–90L14.1*; 1.4×10^−7^
Burdon KP et al. *Diabetologia* 2015 (37); Illumina Human OmniExpress	Fundus exam and modified ETDRS criteria	2	Sight-threatening DR ***^a^***; White Australian: 336	DM2 w/o DR or minimal DR; White Australian: 508	Near *PTK7*; 2.66×10^−7^
Graham PS et al. *BMCMedGenet* 2018 (38); Illumina Human OmniExpress	Fundus exam and EDTRS criteria	2	1 DME; White Australian: 270 2 PDR; White Australian: 176	1 No DR; White Australian: 435 2 No DR; White Australian: 435	1 *MRPL19*; 4.10×10^−6^ 2 Near *LOC285626*; 3.87×10^−6^
Meng W et al. *ActaOphthalmol* 2018 (39); Affymetrix SNP 6.0, Illumina Human OmniExpress	Fundus exam and Scottish Diabetic Retinopathy Scheme or EDTRS or history of laser treatment	2	Severe/proliferative DR ***^b^***; Scottish: 560	DM2 with normal or mild background retinopathy ***^c^***; Scottish: 4106	*NOX4*; 4.05×10^−9^
Pollack S et al. *Diabetes* 2019 (40); Affymetrix SNP 6.0 Illumina 2.5M, 370, 670, Omni Quad, OmniExpress	Fundus exam and ETDRS score	2	***^d^*** Primary: Any DR (≥14) ; AA: 911, EUR: 1,079 Secondary: **1** PDR (≥60); AA: 1,097, EUR: 398 **2** NPDR or worse (≥14); AA: 768, EUR: 644 **3** PDR (≥60); AA: 1,097, EUR: 398	Primary: DM2 w/o DR (<14); AA: 941, EUR: 1,970 Secondary: **1** No PDR (<60); AA: 1,514, EUR 2,848 **2** No DR (<14); AA: 941, EUR: 1,970 **3** No DR (<14); AA: 941, EUR: 1,970	Primary: none Secondary: **1** Near *GOLIM4* for AA; 9.42×10^−9^ Near *NOS2/LYRM9* for EUR; 7.26×10^−9^ **2** None **3** Near *NVL* for EUR; 2.10×10^−9^
Minako I et al. *Hum Mol Genet* 2021; (41) Omni Express Exome Bead Chip. Illumina Human 610K SNP array	Fundus exam and history of laser treatment for DR	2	DM2 with any stage of DR; Japanese: 5532	DM2 with no signs of DR; Japanese: 5565	*STT3B*; P = 1.62 × 10^−9^ *PALM2*; P = 4.19 × 10^−8^ *EHD3*; P = 2.17 × 10^−6^

a severe NPDR, PDR, clinically significant macular edema.

b R3 and R4 on Scottish Retinopathy Scheme.

c R0 and R1 on Scottish Retinopathy Scheme.

d values given are based on EDTRS scale.

AA, African American.

EUR, of European descent.

In 2015, the Burdon study involved 336 cases (with “sight-threatening” retinopathy, defined as NPDR, PDR, or clinically significant macular edema) and 508 controls (participants with at least 5 years of diabetes without DR) in a white Australian cohort. The study did not identify any single nucleotide polymorphisms (SNPs) of genome-wide significance. However, through a meta-analysis combining the discovery cohort and replication cohorts (including type 1 and type 2 diabetes cohorts in London and type 2 diabetes in India), the investigators identified a variant with genome-wide significance on chromosome 17q25.1 (P=4.15×10^-8) (37). In 2018, Graham et al. re-analyzed data from the Burdon study and conducted a GWAS. The study used two independent case-control designs: the first included 270 patients with DME and 435 controls without retinopathy, and the second included 176 patients with PDR and 435 controls without retinopathy. The researchers chose this design because they thought DME and DR might have different genetic mechanisms. The results differed significantly from the previous study, with the most significant SNPs located in different chromosomal regions, although SNPs with genome-wide significance could not be identified. The most significant SNPs were near the MRPL19 and LOC285626 genes on chromosomes 2 and 5, unlike the 2015 study near the PTK7 gene on chromosome 6 (38).

The Genetics of Diabetes Audit and Research (GoDARTS) project has provided valuable insights into the genetic background of diabetic retinopathy. One study analyzed case-control data from a Scottish type 2 diabetes cohort and found that variants in the NOX4 gene, as well as two nearby SNPs (single nucleotide polymorphisms), were associated with severe background retinopathy, including PDR. These variants reached genome-wide significance in this cohort. However, when these findings were used in a meta-analysis with other white cohorts, these SNPs did not reach genome-wide significance thresholds in the replication cohort (39). This phenomenon suggests that certain genetic variants associated with diabetic retinopathy in preliminary studies may not replicate or be consistent across cohorts and populations. This inconsistency in genotype-phenotype relationships across populations may reflect differences in genetic background, environmental factors, and phenotypic expression. It emphasizes the importance of replication and validation across ethnicities and regions when conducting genome-wide association studies. In addition, NADPH oxidase 4, encoded by the NOX4 gene, generates reactive oxygen species intracellularly and participates in the oxidative stress response, which is closely related to the mechanism of diabetes-induced chronic complications, including retinopathy. Although NOX4 and its associated variants presented potential genetic associations in initial studies, the failure of replication in other populations still emphasizes the complexity of genetic studies in different populations.

In 2019, Pollack et al. obtained promising results in a large-scale GWAS study. The study involved 15 discovery cohorts totaling 3,246 subjects of European descent and 2,611 subjects of African American descent, with a large replication cohort of 18,545 Europeans, 16,453 Asians, and 2,710 Hispanics. The study set stricter significance thresholds: for the African American or multiracial cohort, the genome-wide significance criterion was P<3.75×10-^9^; for the European-origin cohort, it was set at P<6.25×10-^9^ (40). In addition, the study considered covariates such as diabetes duration, glycemic control, and race. By performing a Meta- analysis of 1990 cases of diabetic retinopathy (DR) (all manifestations) versus 2911 controls without DR, the study found that no significant polymorphisms were found in either the European or African American descent cohorts. In addition to this, three secondary case- control studies were conducted for subsets of DR, specifically 1) PDR versus no PDR, 2) NPDR or more severe forms versus no DR, and 3) PDR versus no DR. In a meta-analysis of the DR group (PDR versus no DR) in subjects of European descent, a SNP was identified that reached genome-wide significance: rs142293996, located in the intronic region of the NVL gene (P value 2.1 × 10-^9^). Although subsequent replication studies showed that the polymorphism still had the same direction of effect, it failed to reach the genome-wide significance threshold. In the African American discovery cohort, the major finding in the PDR vs no PDR cohort was rs115523882 located near the GOLIM4 gene, a SNP that, although close to the significance threshold (P = 4.1 × 10-^6^), failed to meet the significance criterion, and a replication study was unable to validate this result. In addition, the researchers noted that they failed to replicate some of the results mentioned in other GWAS studies. In addition, Pollack et al. assessed whether polymorphisms clustered in biological networks of proteins or signaling pathways by using the Disease Association Protein-Protein Link Evaluator (DAPPLE) and Meta-Analysis Gene-Set Enrichment of variaNT Associations (MAGENTA) software. In the African American PDR case group, a statistically significant network was identified that contained genes associated with inflammation and protein products known to be highly expressed in ocular tissues. The authors concluded that this network analysis helped identify involved pathways that may not be revealed in a single SNP analysis. The study is considered to be the most robust genomic study to date, given its large sample size, multiple ethnicities, well-defined cases and controls, and adequate adjustment for covariates, as well as repeated validation of previous findings.

A recent Japanese study analyzed data from 11,097 patients with type 2 diabetes mellitus (T2DM), including 5,532 patients with diabetic retinopathy (DR) and 5,565 patients with diabetic nephropathy or diabetic controls without retinopathy, through a genome-wide association study (GWAS). The study identified two new single nucleotide polymorphism (SNP) loci and a new DR susceptibility gene, EHD3. Although this study provides new clues to the genetic risk of DR, the findings have poor replication. Although numerous potential genetic markers were identified, they have failed to be consistently validated in other cohorts or studies, suggesting challenges in studying the genetic mechanisms of DR. Gene-disease associations may be affected by various factors such as population differences, environmental factors, study design, and sample size. Future studies may require larger multicenter collaborations, validation in different ethnic groups, and more precise definition of disease phenotypes to further validate and understand the association between these genetic markers and DR. In the meantime, the use of more nuanced biomarker or functional studies may help reveal how these newly identified genetic variants drive disease progression by affecting cellular and molecular pathways (41).

#### 2.2.3 Whole exome sequencing

Whole exome sequencing (WES) is a cost-effective method with the potential to identify genetic mutations beyond those discovered through GWAS. The exome represents only 1% of the human genome but encompasses 85% of the body’s genetic information. WES research in ophthalmology has been explored in a limited fashion to date, with two studies focusing on diabetic retinopathy (DR). The first study, completed by Shtir et al. in 2016, used an extreme phenotype of diabetes to compare 43 individuals who had diabetes for at least 10 years but did not develop DR (case group) with 64 individuals who developed DR within 10 years (control group) (42). The study aimed to identify variants protective against DR and identified potential candidates including NME3, LOC728688, and FASTK. This suggests that extreme phenotyping methods can help identify disease-modifying variants in DR patients with smaller sample sizes, although further validation is required. In 2017, a second study conducted by Ung et al. used an extreme phenotyping approach to compare 43 patients with PDR requiring surgical vitrectomy (case group) with 13 patients who did not develop DR after 10 or more years of diabetes (control group). These subjects were from the African American Proliferative Diabetic Retinopathy Study (AAPDR), as well as from the Mixed Ethnicity (ME) patient population at the Massachusetts Eye and Ear Infirmary and the Dean McGee Eye Institute at the University of Oklahoma. The study focused on variants present only in the case group and excluded those present in the control group with a minor allele frequency of less than 0.1% and potential protein function impact (e.g., missense variants, loss-of-function variants). Genes had to occur at least 3 times in the AAPDR cohort or at least 2 times in the ME cohort. Ultimately, 44 genes were identified, with 19 containing 25 new polymorphisms. Some of these variants occurred in genes with known functions in angiogenesis (vascular endothelial growth factor-B) and low-density lipoprotein (LDL) cholesterol metabolism (apolipoprotein B), which are strongly associated with PDR development (43). Although WES use for DR research remains limited, these two studies show promise for future research directions.

## 3 Transcriptomics

Transcriptomics is the link between the genome and proteome, utilizing next-generation high-throughput sequencing technology to detect the sum of all RNAs transcribed from a specific tissue or organ under certain conditions. Single-cell RNA sequencing (scRNA-seq) is a novel technology invented by Tang et al. (44), first applied to ophthalmology by Macosko et al. (45) in 2015. It is a current research hotspot. scRNA-seq sample sources include the retina, fibrovascular membranes (FVM), and peripheral blood mononuclear cells (PBMC). In 2020, Van Hove et al. (46) established the first single-cell transcriptional atlas of the retina, linking DR-associated risk genes to cell type-specific expression patterns. They analyzed networks of differentially expressed genes in neural cells, glial cells, and immune cells in DR models. It was found that the differentially expressed genes in optic rod, optic cone, bipolar, and macroglial cells were mainly involved in cellular metabolism, ribosomal gene expression, neuroglial proliferation, immune system activation, and dysregulation of metal ions and redox homeostasis. The results suggest that the macroglia play a key role in the early neurodegenerative process in DR, but the study failed to analyze Müller cells and astrocytes separately.

In a seminal review (47), researchers systematically outlined four key applications of scRNA-seq in unraveling DR pathogenesis: (1) detection of differentially expressed genes (DEGs), (2) identification of critical cellular subpopulations and their transitional states, (3) exploration of intercellular communication networks, and (4) integration with genome-wide association studies (GWAS) to correlate cell types with genetic risk loci. This review further cataloged scRNA-seq-validated DEGs with established functional roles in DR. By enabling high-resolution gene expression profiling, scRNA-seq transcends traditional bulk sequencing to uncover novel disease-associated cellular subtypes and dynamic trajectories, offering unprecedented insights into pathogenic mechanisms and therapeutic targeting.

### 3.1 Microglia

Microglia, the resident macrophages of the retina, are central to early inflammatory responses in DR. Under hyperglycemic or hypoxic conditions, these cells undergo necroptosis to release fibroblast growth factor 2 (FGF-2), driving pathological angiogenesis. He et al. (48). performed scRNA-seq on oxygen-induced retinopathy (OIR) mice, identifying a microglial subpopulation enriched in necroptosis-associated genes RIP3 and MLKL. Hypoxia-triggered necroptosis in this subpopulation promoted FGF-2 secretion, which directly activated retinal endothelial cells (ECs) to induce neovascularization.

Hu et al. (49) generated a comprehensive cellular atlas of fibrovascular membranes (FVMs) from proliferative DR (PDR) patients, highlighting microglia as a dominant population. Pseudotemporal trajectory analysis revealed a pro-fibrotic GPNMB+ microglial cluster originating from retinal-resident microglia (not monocyte-derived macrophages), with marked enrichment of osteopontin (SPP1). This finding aligns with animal studies by Liu (50) and Bai (51), where OIR microglia exhibited upregulated Igf1 and Spp1. Pharmacological inhibition of these genes significantly attenuated pathological neovascularization. Paradoxically, Luo et al. (52) observed that only a minor subset of activated Spp1+/Igf1+ microglia co-expressed thrombospondin-1 (Tsp-1) and fibronectin (Fn1), which suppressed angiogenesis via the Tsp-1/miR-27a-5p exosome/Smad3 axis. These findings underscore the functional heterogeneity of microglia in vascular regulation.

Lv et al. (53) mapped the single-cell transcriptome of streptozotocin (STZ)-induced diabetic retinas, identifying microglia as primary sources of interleukin-1β (IL-1β) and tumor necrosis factor (TNF). Kyoto Encyclopedia of Genes and Genomes (KEGG) pathway analysis revealed metabolic reprogramming (glycolysis, purine metabolism, and triacylglycerol synthesis) within inflammatory microglial subclusters. Ben et al. (54) further described an immunoregulatory microglial subtype enriched in STZ-DR models, characterized by upregulated MAPK, JAK/STAT, and IL-17 signaling. Targeting this subpopulation attenuated retinal inflammation and early DR progression. Strikingly, ligand-receptor pairing analysis in diabetic mice revealed enhanced Csf1r-Csf1 crosstalk between ECs and microglia, driving MAPK-mediated pro-inflammatory cytokine secretion and angiogenesis via paracrine feedback.

Xiao et al. (55) found that microglia were the most sensitive cell type to hyperglycemia and had the highest number of differentially expressed genes when they performed single-cell RNA analysis of retinas from a crab-eating monkey model of spontaneous type 2 diabetes mellitus (T2DM). In a hyperglycemic environment, TNF-α mediates microglia activation through autocrine secretion. Activated microglia not only affect neuronal function by secreting proinflammatory factors, but may also interact with retinal vascular endothelial cells to disrupt the blood-retinal barrier, leading to retinal vascular injury. This finding reveals the critical role of microglia in DR and provides new targets for further therapeutic strategies.

Collectively, scRNA-seq elucidates the dual roles of microglial subsets in DR pathogenesis, positioning them as therapeutic targets for modulating angiogenesis and inflammation.

### 3.2 Endothelial cells

In DR progression, endothelial cell (EC) dysfunction is a central driver of vascular pathology, as evidenced by pathologic dilatation, leakage, and structural destruction of capillaries, and triggers abnormal neovascularization. Molecular mechanism studies have shown that activated ECs promote leukocyte-endothelial adhesion through high expression of inflammatory mediators such as intercellular adhesion molecule-1 (ICAM-1) and synergistic effects with vascular endothelial growth factor (VEGF), which together exacerbate vascular hyperpermeability and pro-angiogenic signaling. It is worth noting that the current study has not fully elucidated the mechanism of EC subpopulation heterogeneity in the pathological process, and it is urgent to reveal the differences in the response of different EC subpopulations to hyperglycemic stimuli and their specific molecular pathways mediating the inflammatory cascade and pathological angiogenesis by single-cell resolution technology.

Studies using scRNA - seq have generated a comprehensive transcriptional profile of retinal cells in the DR. yao et al. (56) identified a unique endothelial cell (EC) cluster that is present only in the diabetic retina of db/db mice and highly expresses Icam-1 and Vcam-1. In addition to pathways associated with inflammation and cell migration, this unique EC cluster also specifically expresses ceramidase-like cellular markers involved in sphingolipid metabolism, such as Acer2 and Plpp1. Subsequent experiments verified the role of Acer2 in pro-angiogenesis and found that ACER2 synergistically inhibited VEGF- induced EC permeability when combined with ranibizumab. Do ng et al. (57) further analyzed the effects of high glucose on retinal vascular endothelial cells by using transcriptomics. The results revealed that the expression of TGF-β pathway members bone morphogenetic protein 4 (BMP4) and SMAD family member 9 (SMAD9) was elevated. Validated by *in vitro* and *in vivo* experiments, the study showed that BMP4 significantly upregulated the expression of SMAD9 and promoted the expression of VEGF (vascular endothelial growth factor) and fibrosis factor. Therefore, the researchers concluded that BMP4 may serve as an important therapeutic target in the course of DR with dual roles of anti-VEGF and anti-fibrosis, which may provide new ideas for the treatment of DR.

Crespo-Garcia et al. (58) found that pathological vasculature is involved in pathways associated with cellular senescence compared with healthy vessels, while inhibitors of the anti-apoptotic protein BCL-xL effectively inhibited pathological angiogenesis. More interestingly, single-cell analysis showed that a population of vascular endothelial cells characterized by senescence and specifically expressing Col1a1 was no longer detected in BCL-xL inhibitor-treated retinas, suggesting that the drug was able to selectively remove senescent cells and thus inhibit neovascularization. Notably, the transcriptomic profile of the Col1a1+ vascular endothelial cell gene cluster was highly homologous to senescent human retinal microvascular endothelial cells, a finding that suggests the study may have cross-species applications. The ability of senescent cell scavengers to effectively remove senescent cells without damaging healthy tissues is a property that suggests great potential for the treatment of diabetic retinopathy (DR). In addition, Bai et al. (59) analyzed scRNA-seq data from STZ-induced diabetic mice and OIR mice and found that the G protein subunit αi2 (Gαi2) was universally expressed in all cell types but significantly elevated in endothelial cells (ECs), suggesting that it may play an important role in ECs. Subsequently, the role of Gαi2 in promoting pathologic angiogenesis in the retina of DR mice through activation of the transcription factor NFAT was experimentally verified.In addition, cellular senescence is closely associated with the pathogenesis of diabetic retinopathy (DR). Single-cell analyses of 85 db/db mice, Akimba mice, and OIR mice all showed enrichment of senescence gene signatures in vascular endothelial cells (ECs), particularly upregulation of the senescence biomarker p53 (58, 60, 61).

Through high-resolution single-cell RNA sequencing, researchers were able to deeply analyze the cellular and molecular mechanisms of diabetic retinopathy. This technology not only successfully identified a subpopulation of endothelial cells with atypical transcriptional features and active angiogenesis, but more importantly, it provides a key molecular target and theoretical basis for the development of breakthrough therapeutic strategies.

### 3.3 Pericytes

As a core component of the neurovascular unit, retinal pericytes play a key role in regulating the structural and functional homeostasis of the blood-retinal barrier and finely regulate vascular development and maturation through paracrine signaling networks. Existing studies have focused on the microvascular degenerative changes triggered by the progressive loss of pericytes in early DR, and the pathological mechanisms underlying their pro-angiogenic phenotypic transformation in proliferative DR have not been fully elucidated. Recent evidence suggests that residual pericytes may synergize pathological neovascular sprouting through matrix remodeling protein secretion with proinflammatory mediator release, but the specific molecular switches and intercellular communication patterns of this process still need to be systematically resolved. Xia et al. (62) identified a subpopulation of pericytes through the establishment of a mouse model of oxygen-induced retinopathy (OIR) and a single-cell transcriptome profiling of the normal mouse retina. This subpopulation was characterized by its significantly high expression of Col1a1 and other genes associated with extracellular matrix (ECM) remodeling. This pericyte subpopulation was significantly enriched in OIR retinas compared with normal control retinas, suggesting its important role in retinopathy. Further experiments demonstrated that Col1a1 expression levels were increased in pericytes of OIR retinas. By silencing Col1a1, the researchers found that this manipulation inhibited angiogenesis and the conversion of pericytes to myofibroblasts during capillary remodeling. These results suggest that Col1a1 may influence the process of angiogenesis during OIR by regulating the remodeling of the extracellular matrix, which promotes retinal vascularization and pericyte transformation. This study reveals the important role of pericytes in retinal vasculopathy and highlights the potential regulatory function of Col1a1 in neovascularization and vascular remodeling. A deeper understanding of these mechanisms may in the future provide new targets and strategies for the treatment of related ocular diseases, including diabetic retinopathy. These findings provide important insights into the molecular mechanisms underlying pericyte dysfunction in DR.

### 3.4 Müller cells

Müller cells are the major glial cells in the retina and extend throughout the retina, from the inner border membrane to the outer membrane. They are not only the structural scaffold of the retina, but also play key roles in metabolic regulation, ionic homeostasis, signaling, and neuroprotection. In DR, functional abnormalities of Müller cells are closely associated with disease progression, exhibiting dual roles of protective and pathogenic properties. Niu et al. (63) found that changes in Rlbp1 expression showed an opposite trend in Müller glial cells versus other retinal cells in db/db mice by scRNA-seq studies. Although Rlbp1 was shown to be upregulated in other cell types, it was downregulated in Müller glial cells, which may be one of the reasons explaining why previous studies have had difficulty in clarifying its changes in diabetic retinopathy (DR). Further, after selective overexpression of Rlbp1 in diabetic Müller glia, it was found that gliosis was suppressed and retinal capillaries and neurons were effectively protected. This suggests that Rlbp1 supplementation may be a promising therapeutic strategy to alleviate DR- related dysfunction.

Single-cell RNA sequencing (scRNA-seq) studies have revealed the complex regulatory mechanisms of Müller glial cells in diabetic retinopathy (DR). Under diabetic pathology, vascular endothelial growth factor A (VEGFA) expression is significantly elevated in retinal ganglion cells (RGCs), optic rod cells, and optic cone cells, whereas Müller glial cell expression levels of its receptor, VEGFR2, are markedly downregulated. This receptor-ligand expression imbalance highlights a unique molecular adaptation strategy of Müller cells in response to the high-glucose microenvironment. scRNA-seq not only captured the dynamic changes in the gene expression profiles of Müller cells themselves (e.g., metabolism-related pathway remodeling), but also resolved their interaction networks with neurons and vascular endothelial cells (e.g., through TSP-1-mediated paracrine signaling). Notably, this cell-specific response pattern provides a key molecular basis for the development of novel therapies targeting the functional regulation of Müller cells (e.g., restoration of VEGFR2 signaling homeostasis or intervention in cell-to-cell communication), which may be expected to alleviate the progression of retinal damage in DR.

### 3.5 Photoreceptors cells (rods and cones)

Photoreceptor cells serve as core components for the conversion of light signals to neuroelectric signals, and their degeneration is an important cause of late vision loss in DR. The application of scRNA-seq in recent years has provided high-resolution insights into the molecular mechanisms of degeneration by deeply resolving the perturbation of gene expression networks in photoreceptor cells by hyperglycemia and ischemic microenvironments. Han et al. (64) established a model of progressive diabetic retinopathy (DR) by using pdx1+/- mutants and glucose-treated adult zebrafish. In moving from wild-type zebrafish to the pdx1+/- mutant to the glucose-treated pdx1+/- mutant, the zebrafish showed elevated blood glucose levels and shortening of the optic cone cells. Single-cell analysis revealed that optic cone cells, the most susceptible type of retinal neurons, underwent three distinct states on a pseudotemporal trajectory. Corresponding to the progressive damage of the optic cone cells, there was a gradual decrease in the expression of the hcn1 gene, suggesting that the reduction of hcn1 may be one of the mechanisms of DR optic cone cell damage. Characterization of this state-specific gene expression profile may further reveal its potential role in DR onset and progression. These in-depth studies provide important targets for the development of strategies against photoreceptor degeneration and neuroprotection in DR patients.

### 3.6 Other cells

In addition to retina-resident immune cells (65), circulating leukocytes may also be involved in inflammation leading to DR. Liao et al. (66) collected peripheral blood mononuclear cells (PBMCs) from patients with type 1 diabetes mellitus and constructed a monocyte profile of circulating immune cells associated with DR. It was found that the expression of AP-1 family members was significantly upregulated in T cells, B cells, and monocytes from DR patients compared to non-DR patients or healthy individuals, and that these genes were enriched in pro-inflammatory pathways. Targeting these generic differentially expressed genes (DEGs) could provide a more comprehensive and effective therapeutic effect by acting on a wide range of retinal cells.

In summary, the application of scRNA-seq has led to a comprehensive characterization of the cellular landscape in the diabetic retina. Through these approaches, researchers can comprehensively map the transcriptome, reveal cell-specific gene expression patterns, and are able to identify subpopulations of cells associated with specific phenotypes. These findings provide deeper insights into the underlying pathogenic mechanisms of DR, contributing to precision therapy.

The scRNA-seq technique provides information from a transcriptomic perspective, while other biological regulatory processes before and after transcription are neglected, highlighting the value of utilizing other single-cell omics techniques. However, scRNA-seq data cannot account for the majority of SNPs localized in the non-coding regions of the genome, and therefore a more scientific and comprehensive integration of genomics and transcriptomics is needed. Jagadeesh et al. proposed a framework for inferring the impact of genetic variation on disease through the integration of scRNA- seq, epigenomic SNP-gene profiling, and GWAS statistical summarization effects on underlying cell types and processes. This approach provides a powerful avenue for future research in the field of diabetic retinopathy (DR), and in particular offers new research directions for exploring previously overlooked cell types (67). Skol et al. Joint genomics and transcriptomics studies have confirmed that follicle stimulating hormone gene (FLCN) is a susceptibility gene for DR (68).

With the continuous development and optimization of the technology, researchers are expected to reveal more potential cellular subpopulations and microenvironmental factors, which in turn will shed light on the complex pathogenesis of DR. Particularly in cellular senescence, immune response and neurodegenerative changes, single-cell sequencing can provide an unprecedentedly precise view, offering important clues for exploring key nodes in the pathological process. In addition, combining multi-omics data (e.g., proteomics, metabolomics, etc.) with the application of artificial intelligence technology, it is expected to achieve early diagnosis, precise typing and personalized treatment strategies for DR in the future.

## 4 Proteomics

Proteomics refers to the entire proteome expressed in the genome of a cell or tissue, i.e., the analysis of the composition, expression level, post-translational modification status, and interactions among proteins of a cell or tissue dynamics from a holistic point of view, in order to understand the development of diseases. The identification and quantification of proteins, including their isoforms, variants, and post-translational modifications, in healthy and diseased ocular tissues has been termed “proteomics” of intraocular fluids (69). Proteomics techniques typically include steps such as protein extraction, separation, MassSpectrometry (MS), and data analysis. In the study of diabetic retinopathy, proteomics techniques have been successfully applied to the analysis of different biological samples to explore protein markers associated with DR. **Table 2** summarizes the potential biomarker proteins of complement found in serum, tear, aqueous humor, and vitreous.

**Table 2:** Potential biomarkers for DR identified in the different parts of the eye.

Serum	Tear	Aqueous humour	Vitreous humour
α-1-acid glycoprotein	β2-Microglobulin	APOA1	APOA1
α-2-antiplasmin	APOA1	Brain-specific angiogenesis inhibitor 1-associated protein 2	APOH
α-2-macroglobulin	Enolase	Cystathionine b-synthase	Complement C3
α-2-HS-glycoprotein	Hsp27	Growth factor receptor-bound protein 10	Complement C4b
APOA1	Immunoglobulin lambda chain	Keratin type I cytoskeletal 9	Complement C9
C-reactive protein	Lipocalin 1	Keratin type I cytoskeletal 10	Complement factor B
C-C motif chemokine 5	Lactotransferrin	MMP-13	Fibrinogen A
Complement factor B	Lacritin	Podocan	Interstitial retinol-binding protein
Complement factor H	Lysozyme C	Serotransferrin	Inter-α trypsin inhibitor heavy chain
E-selectin	Lipophilin A	Selenoprotein P	Pigment epithelial-derived factor
Haptoglobin	Nerve growth factor		Zinc-α2-glycoprotein
Intercellular adhesion molecule 1			
Interleukin-6 receptor			
Pentraxin-related protein 3			
Pigment epithelium-derived			
Plasminogen activator inhibitor 1			
Prothrombin			
Serum amyloid A protein			
Soluble glycoprotein 130			
Stromal cell-derived factor 1α			
Tumor necrosis factor α			
Tumor necrosis factor receptor			
Vascular cell adhesion protein 1			

### 4.1 Serum

Serum is one of the most readily available and accessible biofluids In DR, many serum proteins are altered, including α-2-antifibrinolytic enzyme (SERPINF2), C-reactive protein (CRP), C-C chemokine 5 (CCL5), intercellular adhesion molecule 1 (ICAM-1), interleukin- 6 (IL-6), positive pentameric protein-associated protein 3 (PTX3), pigment epithelium-derived factor (PEDF), plasminogen activator inhibitor 1 (PAI-1), serum amyloid A (SAA), soluble endothelial molecule-1 (soluble e-selectin), stromal cell-derived factor 1 alpha (CXCL12), tumor necrosis factor alpha (TNF-α), vascular adhesion molecule 1 (VCAM-1), and vascular endothelial growth factor (VEGF) (70–80). Among them, VEGF is one of the most important biomarkers associated with DR, and its expression is promoted by retinal ischemia triggered by dyslipidemia and leukocyte aggregation and initiates neovascularization.

Kim et al. validated a set of 27 biomarkers of mild NPDR, including apolipoprotein A-I (APOA1), alpha-2 macroglobulin (A2M), complement factor H (CFH), and thrombospondin (F2), by multiple reaction monitoring (MRM) analysis. Among these proteins, the team assembled a panel of four protein biomarkers, including afamin (AFM), apolipoprotein C-III (APOC3), complement factor B (CFB), and human tissue kinin- releasing peptide inhibitory enzyme-binding protein (SERPINA4), which was able to differentiate between diabetic and nondiabetic patients with an accuracy of approximately 85-100% (81).

One study found that serum levels of five inflammatory proteins were significantly higher in patients with DR than in type I diabetic patients without DR. These 5 proteins are C-reactive protein (CRP), ICAM-1, soluble glycoprotein 130 (sgp130), TNF receptor I, and VCAM-1. Elevated levels of any of these proteins were associated with a significantly increased risk of developing DR (82). In another study, the inflammatory regulatory proteins α2-HS-glycoprotein (AHSG), α1 acid glycoprotein (AGP), apolipoprotein A-1 (APOA1), and haptoglobin (HP) were found to be differentially expressed in the serum of patients with NPDR and PDR compared to healthy controls. Specifically, serum levels of α2-HS-glycoprotein were elevated in PDR patients, whereas levels of AGP and APOA1 were reduced (83). However, the downregulation of AGP was not as expected because α1-acid glycoprotein is usually involved in pro-inflammatory responses. In another independent study, the inflammatory response protein azurocidin (AZU1) was elevated in the serum of diabetic patients, especially in patients presenting with diabetic complications such as retinopathy (84). This protein is thought to play an important role in the regulation of vascular permeability in the retina.

### 4.2 Tears

Tears are an excellent non-invasive sample, and the tear proteome was first applied to DR by Herber in 2000 (85), after which many research groups have studied the protein composition of tears in greater depth, and to date, more than 1,500 tear proteins have been identified (86–88). The generalized decrease in protein content observed at the onset of DR (89) may be due to defective tear formation or more dilute tears Since tears do not come into direct contact with the retina, the use of tears as a source of biomarkers for DR is questionable. It is not clear whether there is a direct causal relationship between changes in proteins in tears and retinopathy, but studies have shown that there is a correlation between the progression of PDR and changes in certain proteins in tears. For example, levels of proteins such as nerve growth factor, APOA1, lipocalin 1, lactoferrin, and lysozyme C are significantly elevated in the tears of patients with PDR, whereas in the tears of patients with non-proliferative diabetic retinopathy (NPDR), there are reduced levels of lipocalin 1, hsp27, and β2-microglobulin and elevated levels of endothelin and neuron-specific enolase. Changes in these proteins provide potential markers for early diagnosis of diabetic retinopathy (89–93).

In addition, a study identified five candidate biomarkers including lipid carrier protein 1 (LCN1), Lacritin protein (LACRT), lysozyme C (LYZ), lipophilin A (SCGB1D1) and immunoglobulin λ chain (IGLC1). These biomarkers, co-expressed with other proteins and combined with clinical images of the retina, were used to develop a machine learning-based diagnostic model (89, 94–96).

### 4.3 Aqueous humor

Aqueous humor(AH) is an integral component of many ocular health functions, and its main components are proteins, water, and electrolytes that play a crucial role in maintaining homeostasis in the anterior eye segment. In a recent study on the proteomics of AH in patients with PDR, LC-mass spectrometry analysis identified 10 proteins associated with PDR that are involved in a variety of biological processes including inflammation, such as apolipoprotein A-I (APOA1), apolipoprotein A-II (APOA2), apolipoprotein A-IV (APOA4), alpha-1-acid glycoprotein 1 (ORM1), etc. This finding suggests a retinal inflammatory response in patients with PDR in which pro-inflammatory cytokines enter the AH through the vascular system or the vitreous (97). Another study comparing the AH of DR patients who underwent cataract surgery with that of non-diabetic patients identified 11 differentially expressed proteins by two-dimensional difference gel electrophoresis(2D-DIGE) and MALDI-TOFMS(matrix-assisted laser desorption/ionization time of flight tandem mass spectrometry) analysis, three of which were associated with inflammation: apolipoprotein A-I (APOA1), selenoprotein P(SELENOP) and cystathionine β-synthase (CBS). Selenoprotein P plays an important role in maintaining oxidative homeostasis and is significantly down-regulated in aqueous humor water in DR patients (98). Cystathione β-synthase, which is highly upregulated in DR patients, is responsible for hydrogen sulfide synthesis and is associated with inflammation and apoptosis (99). Another study comparing AH proteins in the DR and control groups showed that APOA1, serum transferrin, keratin type I cytoskeleton 9, keratin type I cytoskeleton 10, growth factor receptor-binding protein 10, brain-specific angiogenesis inhibitory factor 1-associated protein 2, and cystathionine β-synthase were upregulated in the DR group, whereas matrix metalloproteinase 13, podocan and selenoprotein P were downregulated. In addition, they found that the AH had higher total protein concentrations than patients without DR, which is analogous to findings in vitreous humor (100, 101).

### 4.4 Vitreous humor

The vitreous humor is adjacent to the retina and may reflect changes in the retina. It has been shown that the total protein content in the vitreous of DR patients is higher than in non-diabetic and control samples (101). In a comparative proteomics study, the protein composition of the retina and vitreous was analyzed and showed that these proteins functionally interact in controlling oxidative stress, immune responses, and intracellular protein exchange between the retina and vitreous (102).

Vitreous humor proteins were studied in patients with PDR and non-diabetic patients with macular holes using DIGE and mass spectrometry. Upregulation of zinc-alpha2- glycoprotein, adiponectin (APO) A1, APOH, fibrinogen A, complement factors C3, C4b, C9, and factor B, and downregulation of pigment epithelium-derived factor, mesenchymal retinol-binding protein, and meso-alpha-trypsin inhibitor heavy chain were observed (103). Their other researchers have also observed elevated levels of APOA1 and APOH in patients with PDR, which may lead to nutritional disorders and predispose to disease (104).

Balaiya et al. used proteomics to analyze the vitreous humor of patients with PDR and those with pre-retinal or macular tears. 16 unique proteins were identified in the vitreous humor of patients with PDR, and these proteins functionally related to coagulation, complement, and the kinin-releasing enzyme-kinin system, and may be biomarkers of PDR. The proteins that were differently expressed in the aqueous humor and vitreous of patients with PDR were similar to those of the vitreous humor of the patients with PDR. The reason may be that the vitreous proteins break through the vitreous-aqueous humor barrier or the blood-aqueous humor barrier and penetrate into the aqueous humor (97). In a study comparing the protein expression in aqueous humor of PDR patients with that of age-related cataract patients, a total of 191 proteins were found to be altered, of which 111 were down-regulated and 80 were up-regulated, and the differentially expressed proteins were mainly enriched in the complement and coagulation cascades, the PI3K/Akt signaling pathway, and cholesterol metabolism pathway (105). Weber et al. applied proteomics to analyze the protein alterations and pathway analysis of vitreous humor from patients with PDR and patients with macular tear or anterior retinal detachment, and the results confirmed that vitreous humor from patients with PDR showed significant activation of metabolic pathways and inhibition of neuroprotective pathways (106).

Several studies have also used vitreous proteomics to examine the effect of anti- VEGF vitreous cavity injections on disease progression. Several studies have used vitreous proteomics to analyze the effect of anti-VEGF vitreous cavity injections on disease progression. Although Loukovaara et al. found an increase in complement, coagulation factors, and other inflammatory proteins in the vitreous cavity of DR patients, they failed to determine a significant effect of the anti-VEGF drug bevacizumab on these proteins (107). Interestingly, Wei et al. observed an increase in complement factors, coagulation factors, apolipoproteins, and immunoglobulins after vitreous cavity injections, while photocoagulation was able to reduce levels of the pro-inflammatory protein osteopontin(SPP1) (108). Zou et al. on the other hand compared the proteomes of the vitreous cavities of patients with DR treated with the anti-VEGF drug ranibizumab, and found a decrease in VEGF levels as expected and a Acute inflammatory response, platelet degranulation and complement activating proteins were reduced (109, 110).

Overall, the application of multi-sample proteomics in DR research will greatly contribute to the innovative development of the field and open up new horizons for early diagnosis, precision treatment and personalized management of diabetic retinopathy.

## 5 Metabolomics

Metabolomics is an important branch in the field of “omics” science after genomics, transcriptomics and proteomics, which combines high-throughput analytical techniques with bioinformatics. Therefore, metabolomics provides new ideas for the early diagnosis of diabetes and its complications, and a powerful platform for the discovery of novel markers and new biochemical processes. The Human Metabolome Database currently lists 114,100 metabolite entries, which are far fewer than the number of proteomes, gene variants, and RNA transcripts identified to date, but the diversity of metabolite chemistries and isoforms continues to make this type of research an enormous challenge. In the last 15 years, there has been a growing body of research on DR metabolomics, with the surge in studies focusing on the analysis of vitreous and blood samples. In this review, we focus on summarizing the main findings of DR metabolomics studies on different samples including plasma, serum, vitreous humor, and aqueous humor.

### 5.1 Plasma

Unlike proteomics, most of the mainstream biological research samples for DR metabolomics studies are derived from blood (plasma or serum), mainly due to the fact that blood samples are not volume-limited and are easily accessible. Internationally, DR, characterized by metabolic disorders, is classified into three complex stages: preclinical, NPDR, and PDR, while the classification is slightly different in TCM, which considers DR to be classified into two types of evidence: non-yang deficiency and yang deficiency. In a 2011 study, researchers collected plasma samples and subjected them to GC-TOFMS. It was found that arachidonic acid, pyruvic acid and eight other metabolites could differentiate between preclinical DR, NPDR and PDR stages. Meanwhile, four metabolites could distinguish two TCM syndrome types. Among them, both L-aspartate and pyruvate were recognized as metabolites capable of differentiating between the two stages of DR according to the Western and Chinese medicine syndrome types. However, the abnormal aspartate levels observed in some cases may be due to impaired renal excretion of the amino acid, which needs to be further verified due to the lack of records of chronic kidney disease in this study (111).

Sumarriva et al. used untargeted metabolomics (LC-MS) to analyze plasma and identified 126 and 151 characteristic metabolites between DR and DM and between PDR and NPDR, respectively. In their study, glutamic g-semialdehyde, citrulline, dehydroxycarnitine, and arginine were the key factors contributing to the metabolic alterations between DR and controls. And the combination of glutamate and glutamine was found to improve the specificity of DR versus non-DR in another study (112). Also, carnitine has an important role in distinguishing pathway differences (vitamin D3 metabolism, fatty acid metabolism, etc.) between PDR and NPDR (113).

Sun et al. reported 22 differentially expressed metabolites in a large population- based plasma metabolomics study (DR and PDR) using UPLC-MS and multivariate statistical analyses in which four metabolites, glutamate, N-acetyltryptophan, leucylleucine, and pseudouridine, were singled out to differentiate between PDR and NPDR, and risk score analyses showed that these metabolites were positively Correlation (114).

To further investigate this, Peters et al. performed targeted metabolomic analysis of six arginine- and citrulline-related metabolites. Plasma levels of arginine and citrulline were elevated in DR patients compared to diabetic controls, thus affirming the important role of arginine and citrulline metabolism in DR (115).

Zhu et al. reported changes in fumarate, cytidine, uridine, and acetic acid in a large- scale population-based plasma metabolomics study using LC-MS. According to the researchers, in this study, fumarate was reported for the first time as an abnormal biomarker for the diagnosis of DM/DR, with an area under the curve (AUC) of 0.96. Additionally, cytidine showed excellent performance as a potential marker, with an AUC of 0.95. However, this is not the first time that cytidine has been reported, and as early as in the two studies by Xia et al. (116) and chen et al. (117) cytidine was hypothesized as a potential biomarker for DR (118).

Peng et al. investigated and measured by LC-MS/MS the differences in plasma of twenty alkanes in T2DM patients with and without NPDR. Subsequently, the protective effects of different metabolites (prostaglandin 2a, PGF2a) were tested *in vitro* and *in vivo*. The researchers hypothesized that PGF2a could regulate peripapillary retinal cell migration via FP receptors and reverse some retinal capillary damage, thus providing a protective effect (119).

Some other metabolites, such as pantothenic acid, tryptophan, and combinations of alanine, histidine, leucine, pyruvate, tyrosine, and valine, have also been discussed by researchers, but their specific mechanisms in relation to DR still need to be further clarified (120–122).

### 5.2 Serum

Metabolomics studies based on serum samples have also given us a lot of information. In 2011, Munipally et al. measured for the first time serum samples from DR by reversed-phase high-performance liquid chromatography (detected and quantified by Shimadzu fluorescence detector) with the aim of determining the levels of tryptophan metabolites, and the results (in control subjects, NPDR and PDR patients) showed that the levels of tryptophan metabolites were elevated, but tryptophan itself did not show significant changes (123). Another high-throughput targeted metabolomics study by Yun et al. found that 16 metabolites were common specific metabolites in NPDR and PDR. Among them, total dimethylamine, tryptophan, and total tryptophan were identified as potential contributors to DR progression in DM patients (124). These differences may be influenced by factors such as sample size and different disease groups, and thus need to be further explored.

Curovic et al. identified four metabolites associated with different stages of DR and three triglycerides negatively correlated with DR stages using GC-MS and UPLC-MS studies, respectively. Among them, 3,4 dihydroxybutyric acid was identified as an independent marker of DR progression (125). shen et al. identified 19 metabolites and 13 pathways associated with HE by studying lipidomics and metabolic profiles of patients with HEs (hard exudates) of different severity. A combined model containing 20 lipids including triglycerides, ceramides, and N-acylethanolamine had the strongest discrimination of HEs with an area under the curve of 0.804 (126).

Xuan et al. conducted a multi-platform metabolomics-based study on serum samples, and nearly 300 metabolites (290 and 348) were mainly associated with early DR and DR pathogenesis, respectively. Among them, the biomarker combination of 2- piperidone and 12-hydroxydicarb tetraenoic acid (12-HETE) showed high sensitivity and specificity (0.929 and 0.901) in distinguishing NPDR from NDR over HbA1c (127).

To further understand the metabolic changes from T2DM to DR, Wang et al. conducted a comparative analysis of metabolic profiles at different stages, and they also observed abnormalities such as arginine biosynthesis metabolism, linoleic acid metabolism, and aspartate and glutamate metabolism in an Asian population of DR patients. In addition, this study demonstrated for the first time that serum phosphatidylcholine and 13-hydroperoxyoctadeca-9 (13-hydroperoxyoctadeca-9) and 11-dienoicacid (11-dienoic acid) levels are strongly associated with different stages of DR in patients with T2DM in an Asian population (128).

In a cross-sectional study involving three cohorts from China, Malay and India, 16 serum metabolites associated with DR were identified using NMR technology. Among these metabolites, the investigators found that elevated tyrosine levels and cholesteryl ester-to-total lipid ratios were protective against severe DR, whereas elevated creatinine levels were positively associated with all three DR outcomes (129).

In a score-matched case-control based approach, zuo et al. used uplc-ms-based metabolomics to analyze serum (T2DM and DR). After a series of analyses, the researchers detected 613 specific compounds, 63 of which were significantly associated with the development of DR. Among them, a set of multidimensional network biomarkers containing phenylacetylglutamine, nicotinic acid, linoleic acid, and ornithine were effective in differentiating between DR and T2DM (130). Another similar case-control study also identified some new metabolic changes associated with DR, such as enhanced choline and indole derivatives and diminished trehalose (131).

### 5.3 Vitreous humor

Vitreous metabolomics is considered to be closer to the metabolic profile of the retina. The biochemical changes observed in the vitreous closely reflect alterations in retinal homeostasis, which is of great significance for guiding treatment in the clinic.

Vitreous metabolomic analysis using 1H-NMR was performed by Barba et al. to explore metabolism between non-diabetic patients (NDM) with macular lentigo surgery and type 1 diabetic patients (T1DM) with PDR. Vitreous samples from patients with PDR were characterized by significant deficiencies in ascorbic acid and galactitol, as well as elevated levels of lactate. However, the study has some limitations, as this may make these identified metabolites irrelevant to DR, considering that vitreous hemorrhage is frequent in patients with PDR (132).

However, In the study by wang et al. the authors compared the metabolic profiles of plasma and vitreous. Five metabolites were found to be found to overlap. Specifically, there was a significant increase in phenylacetylglutamine and a significant decrease in valproic acid, which differed from previous findings and which the authors attributed to racial differences (121). Another study by the team also identified potential DR biomarkers in vitreous samples by GC-MS, including myo-inositol, creatinine, uric acid, pyruvic acid, and several amino acids, six of which were selected as new metabolites not found in previous vitreous humor studies. The results of metabolic pathway analysis showed that metabolic pathways such as arginine-proline metabolism and valine-leucine-isoleucine biosynthesis are involved in the pathogenesis of DR (133). And arginine-proline metabolic pathway is considered to be a possible therapeutic target for DR (134).

In addition, purine metabolism has also b been detected in studies of DR. A study by Haines et al. found that the purine metabolite xanthine was a major biomarker in distinguishing DR patients from healthy controls. In addition, proline and citrulline play an important role in differentiating DR from controls and from patients with rhegmatogenous retinal detachment (RD) (135). Recently, Tomita et al. found significant differences in creatine in vitreous humor of PDR patients compared with controls by UPLC- MS, and creatine supplementation inhibited pathological retinal neovascularization in a mouse model (136).

### 5.4 Aqueous humor

In the preclinical phase of DR, where vitrectomy is not indicated and obtaining vitreous samples for analysis is not feasible, aqueous humor has potential utility as an alternative. There are limited metabolomics studies on human aqueous humor samples in the context of DR. Jin et al. performed 1H-NMR analysis of cataract samples (DM-complex cataracts, DR-complex cataracts, and senile cataracts in patients with cataracts) by using 1H-NMR techniques, two-dimensional homonuclear full correlation spectroscopy and two- dimensional pulsed-field gradient correlation spectroscopy. This is the first study to investigate AH in DR through a metabolomics approach, aiming to explore the different metabolic profiles of aqueous humor in DR patients. After a comprehensive series of analyses, the study identified several metabolites with the highest degree of variability. Notably, succinate, lactate, asparagine, histidine, glutamine, and threonine were identified as the most variable metabolites, which may be associated with the progression of DR (137).

In addition, Wang et al. also performed a metabolomics study with aqueous humor water and vitreous samples, and they found that eight metabolites were present in the aqueous humor water samples, and three of these metabolites (citrulline, myo-inositol, and d-glucose) were observed in the vitreous samples as well (133). Another study found that similar to the results of vitreous humor metabolomics analysis, the levels of cystine, CysSSH, and oxidized glutathione trisulfide (GSSSG) were higher in the AH of DM patients than in controls. This result, on the other hand, illustrates the alternative role of vitreous in DR metabolomics analysis (138).

### 5.5 Other samples

To find diagnostic markers for DR, the most logical material to study is the retina, as it is the site of complications in DR. However, this tissue is not readily available in humans, and most retinal studies have been performed in postmortem humans or in animal models. In 2011, Marchetti et al. studied retinal ischemic retinopathy using a combination of metabolomics and molecular biotechnology to explore the underlying mechanisms of DR. In the metabolomics analysis, metabolites that undergo deregulation after hypoxic disposition were analyzed using tandem mass spectrometry, and abnormal metabolites included 7-hydroxycholesterol, 7-ketocholesterol (139).

Lv et al. performed metabolomics, lipidomics, and RNA profiling using BV2 mouse glial cells, which represent the DR microenvironment, to paint a full picture of local metabolic changes and explore the impact of the metabolic microenvironment on immune mechanisms. In the metabolomics section, a total of 78 differential metabolites were identified, which were mainly significantly enriched in purine metabolism, glycolysis and other metabolic pathways (53). However, as the most reasonable sample for identifying DR biomarkers, the metabolomics study of the retina should be thoughtfully arranged, because the time of retinal tissue collection and the method of collection, both of which may have a large impact on the results of the study.

Two other samples commonly used for DR metabolomics studies are urine and feces. Urine, which is easy to collect and rich in metabolites, has become a valuable source for the discovery of non-invasive biomarkers in metabolomics studies (140). Wang et al. set up a urine metabolomics study based on UPLC-Q-ExactiveOrbitrapMS to explore the metabolic changes in DR rats and evaluate the therapeutic effect of Kidney and Blood Formula BP on DR, which identified nine potential biomarkers, which were significantly correlated with tryptophan metabolism and lipid metabolism and intestinal microbial metabolism. These biomarkers were significantly associated with tryptophan metabolism, lipid metabolism and gut microbial metabolism (141). In addition, urinary metabolomic analysis of diabetic model rats showed that metabolites altered by exposure to free CML had effects on metabolic pathways such as carbohydrate metabolism, amino acid metabolism and tricarboxylic acid (TCA) cycle (142).

To explore the relationship between gut microbial metabolism and DR, researchers performed metabolomic analysis of fecal samples using ultra-high performance liquid chromatography-mass spectrometry (UHPLC-MS) and liquid chromatography-mass spectrometry (LC-MS). In the study by Liu et al. untargeted metabolomics analysis was performed on DR and non-DR stool samples from patients with type 2 diabetes. The results of the study showed a significant increase in Acidococcus, Escherichia coli and Enterobacteriaceae bacteria and a significant decrease in Bifidobacteria and Lactobacilli in DR patients. In addition, the percentage of Bartonella was significantly lower (143). In addition, Zhou et al. found that concentrations of niacin, nicotinamide, succinate, and carnosine were significantly lower in DR compared to healthy subjects; six metabolites were decreased and nine metabolites were increased in diabetic patients compared to diabetic patients. KEGG annotation of the metabolomic data revealed that the metabolite composition of 17 pathways differed substantially between DR patients and healthy controls, whereas only 2 pathways differed significantly between DR patients and DM patients (144). Also, Ye et al. demonstrated a significantly different fecal metabolic profile between PDR and NDR, which is enriched in microbial metabolism, and arachidonic acid, which are known mediators in the development of DR. However, these alterations in fecal metabolites may originate from alterations in the gut microbiome in DR that subsequently lead to disease progression, which requires further study (145).

## 6 Integrated multi-omics analysis in DR

Although the above studies of different omics have played an important role in exploring the pathogenesis of DR, providing molecular markers, and searching for therapeutic targets, a single research method often fails to fully explain the full picture of the disease because the onset and progression of DR involves changes at multiple systems and levels. Therefore joint multi-omics analysis can characterize DR more comprehensively from multiple levels and perspectives. For example, by integrating genomics and transcriptomics studies, Skol et al. demonstrated that the follicle stimulating hormone gene (folliculin, FLCN) is a susceptibility gene for DR (68).

More commonly, integration focuses on transcriptomics and proteomics. Mass spectrometry-based single-cell proteomics allows the analysis of more proteins and post-translational modifications without the need for affinity reagents and helps to elucidate signalling pathways essential for single-cell function. Boneva et al. (146) performed the first in-depth single-cell proteomic and transcriptional analyses of tissue samples from diabetic retinal neovasculature with the aim of characterising the relevant cell types. They found a large number of HLA-DR+ immune cells co-expressing α-SMA, suggesting that these cells undergo transdifferentiation to myofibroblasts in retinal neovascularisation (147). Another study at first compared the proteomics of vitreous humor from patients with PDR and those with foramen ovale detachment and found that histone B, D, and L were significantly down-regulated in the vitreous humor of patients with PDR. Further validation of this finding in a high-glucose-induced model of retinal vascular endothelial cells confirmed that down-regulation of histone B, D, and L increased apoptosis by decreasing autophagy (148). In the study by Lv et al. (53), researchers not only used single-cell RNA sequencing to identify a microglial subpopulation with altered metabolic pathways in a DR mouse model but also performed integrated metabolomic, lipidomic, and RNA expression profiling on microglial cell line samples. Multi-omics integration revealed that this microglial subpopulation exhibits a distinct metabolic bias, particularly favoring glycolysis, purine metabolism, and triacylglycerol synthesis. This metabolic reprogramming reflects functional adaptation under inflammatory conditions and is potentially linked to DR progression. Additionally, leveraging metabolomics and transcriptomics, Zhou et al. (149) identified elevated levels of the unique metabolite indoxyl sulfate (IS) in the vitreous humor of DR patients. Integrated RNA-seq analysis further demonstrated that IS induces retinal microvascular damage in DR by upregulating COX-2 expression and PGE2 production.

Furthermore, combined proteomic and metabolomic analysis of serum exosomes by Yang et al. implicated the upregulation of the fibrinogen alpha (FIBA) chain and downregulation of 1-methylhistidine in diabetic endothelial dysfunction, impacting both macro- and microvascular complications. However, further cohort studies are required to delineate the specific roles of FIBA and 1-methylhistidine in the development of DR (150).

As many of the examples above show, studying “omics strategies” can provide important insights, and integrated multi-omics analyses significantly enhance the consistency, coherence, and persuasiveness of experimental findings. However, with the full integration of datasets, it is possible to detect complex interactions that can help deepen our understanding of how networks of “omics factors” work together to interfere with biological pathways and influence disease. Each omics focuses on and resolves specific factors, such as genetic, lifestyle, environmental, inflammatory, co-morbid, or demographic characteristics, to reveal complex interactions and contribute to our deeper understanding of DR pathology and its risk factors.

## 7 Omics-biomarker models

The National Institutes of Health(NIH) Biomarkers Definitions Working Group describes a biomarker as “a characteristic that is objectively measured and evaluated as an indicator of normal biological processes, pathogenic processes, or pharmacologic responses to a therapeutic intervention” (151). For DR, biomarkers can include both imaging and biochemical markers. In terms of imaging biomarkers, structural optical coherence tomography (OCT) is certainly helpful in quantifying certain parameters of the retina, such as central retinal thickness and the presence and characteristics of intra- or subretinal fluid. A new promising diagnostic technique is OCT angiography, but its complete validation in DR is still under debate (152). Circulating (serum) biochemical biomarkers contribute poorly to the understanding and management of DR and diabetic macular degeneration and are not used in clinical practice. This is mainly due to the limited correlation between serum parameters (mainly inflammatory cytokines such as interleukins and VEGF) and diabetes-related retinal complications such as DME, PDR or enlargement of the centro-convex avascular zone. In general, when entering a new area of biomarker research, the process from initial discovery studies to final clinical application can be divided into three steps (153, 154). First, an unbiased approach using an untargeted platform paves the way for new discoveries and hypotheses to be generated. The first step in discovery research is sometimes referred to as a “fishing expedition”-however, this approach is critical to uncovering new pathways that may lead to knowledge about the underlying pathophysiology of the DR and associated biomarkers. Second, a narrowed approach was used to validate important findings from previous studies using targeted methods in independent larger cohorts. Third, biomarkers need to be brought into routine clinical practice. Simple, robust, inexpensive, and high-throughput methods are needed to successfully accomplish this step. In the DR omics study, discovery and validation studies are underway, but clinical applications and breakthroughs have not been established (53). In recent studies, researchers have noted the expression of serum long non-coding RNA (lncRNA) in diabetic retinopathy (DR) patients (155) and have successfully developed a novel DR diagnostic model based on multiple lncRNAs, which demonstrates high accuracy (156). A review by Pang et al. explored the usefulness of artificial intelligence (AI)-assisted integrated multi-omics analysis in the diagnosis and treatment of DR to facilitate the development of individualized medicine (157). To date, no metabolite has been identified to bring it into routine clinical use. However, encouragingly, researchers have noted that multi-omics studies exploring biomarkers can provide deeper insights into the pathophysiological mechanisms of DR, offering innovative treatment approaches (158). There is still a way to go from finding significant associations in discovery studies to understanding causality and the potential for clinical application. In addition, methodological improvements and standardization are needed before implementation in clinical practice.

Many researchers have made it clear that to obtain meaningful results, we must rely on high quality data. They have emphasized that bias, errors, omissions, lack of standardized reporting, and methodological differences must be taken into account, and have called for greater use of standardized references, protocols, and quality control procedures to improve the reproducibility of studies (19, 47, 159–162). In addition, the use of collaborative resources is crucial to advancing DR research.

Nevertheless, many challenges remain in the field of multi-omics. For example, the high cost of omics analysis means that studies can often only be performed on the basis of small sample sizes, which can limit the power of the analysis, resulting in obtaining small p- values or effect sizes and producing limited results. The resolution varies across different omics, and in practice, the level of resolution will be adjusted depending on the budget, such as the depth of reads in transcriptomics or the size of the array panel. Different types of samples, such as blood, urine, tissues, cell cultures, or animal models, can also add complexity and be a source of heterogeneity when datasets are integrated. In addition, histologic studies need to rely on ethnically and socially diverse cohorts to ensure broad applicability of findings. Therefore, researchers should pay special attention to the details of all aspects of sample collection, handling, storage, extraction, and analysis, as even small changes may affect the final results. Collaboration on a global scale promotes more extensive and coordinated research, enhances diversity, drives new discoveries, and increases the general applicability of research. Despite the progress of multi-omics research in discovery studies of DR, its clinical translation still faces many challenges, especially the validation and standardization of biomarkers. Integration of data from different omics, innovation of analysis methods, and improvement of data quality control will be key directions for future research. In addition, the application of artificial intelligence (AI) technology provides new opportunities for the comprehensive analysis and clinical application of multi-omics data, which is expected to promote the development of personalized medicine.

Future work needs to further extend this study to include more diverse data types such as environmental, co-morbid and demographic data. This is important because the impact of environmental exposures, such as pollution, on DR risk has also been identified as a key driver of multimorbidity co-morbidity, with complex interactions between conditions that may influence disease progression. This information will improve our ability to provide personalized medications or predict disease trajectories. In addition, the wide inequalities in eye health and eye disease care Multi-omics studies must incorporate analyses of diverse cohorts to ensure that findings are broadly applicable and improve equity in eye care. In achieving these breakthroughs, improved standardization of research and data quality control will be critical, and collaborative interdisciplinary resources will be an important driver of DR research.

## 8 Conclusion and future perspectives

With diabetes set to grow by nearly 20% globally between 2021 and 2030, there is an urgent need to discover new ways to manage complications such as DR. The future of chronic eye disease treatment will be closely linked to the concept of precision medicine, requiring tailored treatment for patients based on their phenotypic profile. From this perspective, integrating multi-omics studies to explore possible, potential biomarkers will help assist in the early diagnosis of patients with DR and guide clinicians in selecting interventions that will benefit the individual the most. Precision medicine is widely used in oncology, especially in the field of oncology, where biopsies are almost always used to determine the cause of the disease and to select appropriate treatment options. Indeed, direct sampling of the eye by means of a liquid biopsy (including aqueous or vitreous samples) allows for the obtaining of direct biological samples to be studied *in vivo* for the presence and changes in the concentration of specific molecules. This is probably the most rational and accurate method to date, and could also provide new insights into retinal diseases such as DR in the field of ophthalmology (163). Beyond that, validation of the results from single-cohort studies is needed. In addition, the relationship between metabolites and gut microbiota, as well as the effects of factors such as drugs, diet, day- to-day changes and changes over time on the metabolome should be further investigated. More importantly, quantification, automation, reliability and reproducibility between different laboratories and countries must be continuously improved and refined before clinical application of these potential biomarkers. The multi-omics approach provides opportunities for new knowledge generation and discovery of new patterns that can facilitate the discovery of new biomarkers, therapeutic development, or personalized medicines, thus helping to overcome inequalities in the treatment of diabetic retinopathy (DR).

In the future, joint multi-omics analysis will play an increasingly important role in the precision diagnosis, early warning, and personalized treatment of diabetic retinopathy. With the advancement of high-throughput technology and the popularization of artificial intelligence tools, the integration of data from different omics will be more efficient and can reveal more molecular mechanisms of disease occurrence. At the same time, interdisciplinary cooperation and the construction of international cooperation platforms will accelerate the translation of research results, enabling these discoveries to enter the clinical application stage more quickly. Through these efforts, early screening of DR, discovery of therapeutic targets and personalized treatment strategies are expected to be significantly improved.
